# The determinant of health insurance ownership among pregnant women in Indonesia

**DOI:** 10.1186/s12889-021-11577-z

**Published:** 2021-08-11

**Authors:** Agung Dwi Laksono, Ratna Dwi Wulandari, Ratu Matahari

**Affiliations:** 1 The Ministry of Health of the Republic of Indonesia, National Institute of Health Research and Development, Jakarta, Indonesia; 2 Faculty of Public Health, Universitas Airlangga, Surabaya, Indonesia; 3 Faculty of Public Health, Ahmad Dahlan University, Jogjakarta, Indonesia

**Keywords:** Health knowledge, Reproductive health, Maternal health, Pregnancy care, Health insurance, Risk pregnant woman, Public health

## Abstract

**Background:**

Health insurance ownership is one indicator of the readiness of pregnant women for the delivery process. The study aimed to analyze the determinants of health insurance ownership among pregnant women in Indonesia.

**Methods:**

The study population was pregnant women in Indonesia. The study involved 2542 pregnant women in Indonesia. The variables analyzed included type of place of residence, age group, education level, employment status, marital status, parity, wealth status, and know the danger signs of pregnancy. In the final step, the study employed binary logistic regression to explain the relationship between health insurance ownership and predictor variables.

**Results:**

The results show that pregnant women with higher education were 3.349 times more likely than no education pregnant women to have health insurance. Pregnant women with wealth status in the middle category were 0.679 times the poorest pregnant women to have health insurance. Meanwhile, the richest pregnant women had 1.358 times more chances than the poorest pregnant women to have health insurance. Grande multiparous pregnant women were 1.544 times more likely than primiparous pregnant women to have health insurance. Pregnant women who know the danger signs of pregnancy were 1.416 times more likely than pregnant women who don’t see the danger signs of pregnancy to have health insurance.

**Conclusions:**

The study concluded that four variables, including education level, wealth status, parity, and knowledge of the danger signs of pregnancy, were significant determinants of health insurance ownership in Indonesia.

## Background

The use of antenatal care (ANC) is an essential factor in reducing maternal mortality and infant mortality [1]. Maternal mortality is a critical factor in determining the health status of an area. It is not surprising that it is a point of attention in the sustainable development goals (SDGs) program points 3.1 and 3.2 [2]. In Indonesia, the Ministry of Health recommends that pregnant women should have at least four ANC visits during their pregnancy; one visit in the first trimester, one in the second trimester, and two in the third trimester [3]. However, in some developing countries, access to the use of ANC services is still very minimal. Indonesia is a lower-middle-income country with the highest maternal mortality rate in the South-East Asia region [4–6]. Statistically, the maternal mortality rate in 2012 was at 359 cases per 100,000 live births and decreased in 2015 to 305 cases per 100,000 live births [7].

Based on the high maternal mortality rate, the Ministry of Health of the Republic of Indonesia initiated the birth insurance program (Jampersal) as an alternative solution for pregnant women who have economic difficulties paying for ANC services, post-delivery services, and postpartum family planning [8]. However, in 2014 the birth insurance program was replaced with a national insurance program called the National Health Insurance (NHI), which includes insurance services for outpatients and inpatients in health services and several private health services. NHI also has ANC, childbirth, and postpartum services to support universal health coverage goals by 2019 [3]. However, participants must pay a fee to receive the services of the NHI program. Although the government pays this fee for the low-income families through a subsidy mechanism [9, 10].

Several studies explain that the lack of ANC access for pregnant women is related to geographical conditions, including distance to access services and pregnant women’s socioeconomic status [2, 11, 12]. The high cost of services is the most decisive factor that discourages a pregnant woman from accessing ANC [13, 14]. As a solution to this situation, the government implemented a health insurance system as a mitigation measure for ANC services based on the high cost of care [8, 15, 16]. One indicator of the success of the NHIS program implementation will be in the distribution of the wealth status of the recipients. The program was targeted at the poor and the entire community, as insurance ownership is known to be an essential factor in the utilization of ANC services by poor women in urban hospitals [9, 17].

Previous studies in Nigeria have reported that several factors could affect health insurance ownership, including marital status, education level, work type, monthly income [18]. Meanwhile, previous studies in Indonesia found that type of residence, age, wealth status, income, smoking behavior, and history of chronic disease also influenced health insurance ownership [19–21].

Several studies have also shown the positive impact that insurance ownership has on increasing access to ANC services [3, 22–24]. Other studies conducted in Ghana, Indonesia, and Rwanda have also demonstrated that health insurance increases ANC access by 8% in Ghana, 3% in Indonesia, and 11% in Rwanda [2]. However, gaps in the utilization of the NHI program are still visible. Geographic factors related to distance and transportation costs may be responsible. For example, there are more health care facilities in the western than eastern part of Indonesia (Sulawesi to Papua). Low levels of education that is linked with poverty could be additional factors for the poor utilization of health care services [9]. Based on these realities, this study aims to analyze the determinants of health insurance ownership among pregnant women in Indonesia.

## Methods

### Data source

The study was a cross-sectional study. This study was conducted using secondary data from the 2017 Indonesian Demographic and Health Survey (IDHS). The IDHS is a part of an international survey network and part of the Demographic and Health Survey (DHS) series. Internationally, the implementation of DHS is under the control of the Inner City Fund (ICF). The study population was pregnant women in Indonesia. Meanwhile, sampling for The 2017 IDHS used stratified two-stage sampling. Stage 1, selecting several census blocks in a systematic proportional to size probability with the size of the number of households resulting from the 2010 population census listing. In this example, an implicit stratification procedure based on urban and rural regions was used and sorting census blocks based on the wealth index category of the 2010 population census data. Stage 2 picks 25 ordinary households in each census block based on updating the households in each census block [7].

This study’s unit of analysis was pregnant women of childbearing age (15–49 years). The sample size used was 2542 pregnant women.

### Variables

This study’s dependent variable was health insurance ownership divided into two categories: health insurance and not having. Health insurance covers all health insurance types, both those managed by the central government, local governments, and those operated by the private sector. Meanwhile, the independent variables involved in this study’s analysis include the type of place of residence, age groups, education level, employment status, marital status, wealth status, parity, and knowledge of the danger signs of pregnancy.

The type of place of residence consists of two categories, namely urban and rural. The study divided the age group at 5-year intervals into seven groups. The education level consists of four strata, namely no education, primary, secondary, and higher. Employment status consists of two categories, namely unemployed and employed. Marital status consists of three types: never in a union, married or living with a partner, and widowed or divorced. The study determined wealth status based on the wealth index calculation. The wealth index was a composite measure of a household’s cumulative living standard. The study calculated wealth index using easy-to-collect data on a household’s ownership of selected assets, such as televisions and bicycles; materials used for housing construction; and types of water access and sanitation facilities. The wealth index consists of five categories: the poorest, poorer, middle, richer, and the richest [7]. Parity was the number of children who have been born alive. Parity consists of three categories, namely primiparous (≤ 1), multiparous (2–4), and grande multiparous (> 4). Know the danger of pregnancy was the respondent’s knowledge of prolonged labor risks, vaginal bleeding, fever, convulsions, baby in the wrong position, swollen limbs, faintness, breathlessness, tiredness, and others. Knowing the danger of pregnancy consists of two categories: do not know and know. Respondents fall into the “know” category if they admit to knowing all the risks of pregnancy.

### Data analysis

In the early stages, the study carried out a test to ensure no collinearity between variables. Furthermore, the study used the chi-square test for bivariate analysis. In the last step, the study employed binary logistic regression to explains the relationship between health insurance ownership and predictor variables. The research carried out all statistical analyzes by SPSS 22 software.

## Results

Table 1 shows the results for the collinearity test of health insurance ownership among pregnant women in Indonesia. The results of the analysis indicate that there is no collinearity between the dependent and independent variables. The tolerance value of all variables, as shown in Table 1, is more significant than 0.10. At the same time, the VIF value for all variables is less than 10.00. Then referring to the basis of decision making in the test, the study concluded that there were no multicollinearity symptoms in the regression model [25].

**Table 1:** Results for the collinearity test of health insurance ownership among pregnant women in Indonesia (*n* = 2542)

VARIABLES	COLLINEARITY STATISTICS	
	Tolerance	VIF
Type of place of residence	0.736	1.359
Age group	0.722	1.385
Education level	0.774	1.291
Employment status	0.946	1.057
Marital status	0.989	1.011
Wealth status	0.622	1.607
Parity	0.695	1.438
Know the danger signs of pregnancy	0.926	1.080

*Dependent Variable: Health Insurance Ownership

### Sociodemographic characteristics

Table 2 shows the descriptive statistics of health insurance ownership among pregnant women in Indonesia. Table 2 indicates that pregnant women are dominated by those who live in rural areas, whether they have health insurance or not. The two categories of health insurance ownership were most populated by women in the 30–34 age group.

**Table 2:** Sociodemographic characteristics of health insurance ownership among pregnant women in Indonesia (n = 2542)

CHARACTERISTICS	HEALTH INSURANCE OWNERSHIP				***p***-value
	No		Yes		
	n	%	n	%	
**Type of place of residence**					0.055
- Urban	459	44.7	737	48.6	
- Rural	567	55.3	779	51.4	
**Age groups**					**0.006
- 15–19	9	0.9	9	0.6	
- 20–24	91	8.9	108	7.1	
- 25–29	269	26.2	370	24.4	
- 30–34	308	30.0	472	31.1	
- 35–39	284	27.7	430	28.4	
- 40–44	54	5.3	123	8.1	
- 45–49	11	1.0	4	0.3	
**Education level**					***0.000
- No education	23	2.2	17	1.1	
- Primary	315	30.7	407	26.8	
- Secondary	602	58.7	835	55.1	
- Higher	86	8.4	257	17.0	
**Employment status**					**0.005
- Unemployed	632	61.6	849	56.0	
- Employed	394	38.4	667	44.0	
**Marital status**					0.109
- Never in union	0	0.0	3	0.2	
- Married/living with partner	1017	99.1	1507	99.4	
- Widowed/divorced	9	0.9	6	0.4	
**Wealth status**					***0.000
- Poorest	321	31.3	468	30.9	
- Poorer	212	20.7	270	17.8	
- Middle	237	23.1	240	15.8	
- Richer	135	13.1	235	15.5	
- Richest	121	11.8	303	20.0	
**Parity**					**0.009
- Primiparous	282	27.5	415	27.4	
- Multiparous	649	63.3	902	59.5	
- Grande multiparous	95	9.2	199	13.1	
**Know the danger signs of pregnancy**					***0.000
- No	425	41.4	470	31.0	
- Yes	601	58.6	1046	69.0	

Note: ∗ *p* < 0.05; ∗∗ *p* < 0.01; ∗ ∗ ∗*p* < 0.001

Table 2 shows that pregnant women with secondary education lead the two categories of health insurance ownership based on the education level. Meanwhile, based on employment, the two types of health insurance ownership were dominated by unemployed women.

Based on wealth status and parity, the poorest women and multiparous women dominated pregnant women with health insurance. Similarly, there were more pregnant women who knew the danger signs of pregnancy among those with health insurance.

### Determinants of health insurance ownership

Table 3 shows the determinants of health insurance ownership among pregnant women in Indonesia. The test at this final stage is to determine the determinant of health insurance ownership among pregnant women in Indonesia. As a reference, the chosen category was “do not have health insurance.”

**Table 3:** The result of binary logistic regression of health insurance ownership among pregnant women in Indonesia (n = 2542)

PREDICTOR	HEALTH INSURANCE OWNERSHIP			
	***p***-value	AOR	95% CI	
			Lower Bound	Upper Bound
Age group: 15–19	–	–	–	–
Age group: 20–24	0.714	1.201	0.452	3.189
Age group: 25–29	0.710	1.199	0.462	3.109
Age group: 30–34	0.654	1.246	0.476	3.259
Age group: 35–39	0.682	1.225	0.465	3.228
Age group: 40–44	0.256	1.802	0.652	4.982
Age group: 45–49	0.327	.471	0.105	2.119
Education level: No Education	–	–	–	–
Education level: Primary	0.077	1.842	0.937	3.620
Education level: Secondary	0.066	1.884	0.960	3.698
Education level: Higher	**0.001	3.349	1.631	6.877
Employment status: Unemployed	–	–	–	–
Employment status: Employed	0.244	1.109	0.932	1.319
Wealth status: Poorest	–	–	–	–
Wealth status: Poorer	0.272	0.874	0.688	1.111
Wealth status: Middle	**0.002	0.679	0.533	0.866
Wealth status: Richer	0.562	1.085	0.824	1.430
Wealth status: Richest	*0.035	1.358	1.021	1.805
Parity: Primiparous	–	–	–	–
Parity: Multiparous	0.755	0.967	0.782	1.195
Parity: Grande multiparous	*0.014	1.544	1.092	2.182
Know the danger signs of pregnancy: No	–	–	–	–
Know the danger signs of pregnancy: Yes	***0.000	1.416	1.190	1.686

Note: ∗*p* < 0.05; ∗∗*p* < 0.01; ∗∗∗*p* < 0.001

Table 3 shows that four variables are the determinants of health insurance ownership among pregnant women in Indonesia. First, education level. Pregnant women with higher education are 3.349 times more likely than non-educated pregnant women to have health insurance (AOR 3.349; 95% CI 1.631–6.877).

Second, wealth status. Pregnant women in the middle category of wealth status are 0.679 times less likely than pregnant women of poorest wealth status to have health insurance (AOR 0.679; 95% CI 0.533–0.866). Meanwhile, the richest pregnant women had 1.358 times more chances than pregnant women of poorest wealth status to have health insurance (AOR 1.358; 95% CI 1.021–1.805).

Third, parity. Grande multiparous pregnant women are 1.544 times more likely than primiparous pregnant women to have health insurance (AOR 1.544; 95% CI 1.092–2.182). It means grande multiparous pregnant women have a higher chance than the primiparous pregnant women to have health insurance.

Fourth, knowledge of the danger signs of pregnancy. Pregnant women who know all the danger signs of pregnancy are 1.416 times more likely to have health insurance than pregnant women that don’t know all the danger signs of pregnancy (AOR 1.416; 95% CI 1.190–1.686).

## Discussion

The analysis found that pregnant women with higher education had a higher chance of having health insurance than other education levels. This information suggests that higher education provides a better understanding of the benefits of health insurance [19, 26]. On the other hand, a better education level also encourages pregnant women to give birth in health care facilities [27]. A study in South-east Nigeria informed that the main reasons cited by people who do not want to participate in health insurance mechanisms are a poor understanding of how the health care system works and a lack of a steady source of income [18].

Generally, several previous studies have informed that better education levels are positively related to program performance output in the health sector [28–32]. On the other hand, lower education levels are a barrier to achieving better performance in the health sector [33, 34].

The multivariate analysis results found that wealth status is one of the determinants of health insurance ownership among pregnant women. This finding confirms previous studies’ works with the same research theme in Nigeria and Ghana, which informed that those who have good wealth status are more likely to participate in health insurance [18, 35, 36]. Pregnant women with low wealth status probably do not have a fixed source of income. A pregnant woman or her husband has a type of job that is not permanent, making it difficult to set aside income to pay health insurance premiums [18, 20, 37, 38].

The results inform that the grande multiparous pregnant women have a higher probability than the primiparous pregnant women to have health insurance. This information indicates that pregnant women with parity in the grande multiparous category are aware of the increased risk and dangers of having more than four pregnancies. Previous pregnancy experiences can also trigger greater risk in future pregnancies [39, 40]; the chances are for pregnant women and their babies [40, 41].

Finally, pregnant women who know the danger signs of pregnancy have a better chance than pregnant women who don’t know the danger signs. This finding is in line with the previous variable, parity. Better knowledge of pregnancy’s danger signs makes pregnant women more aware and better prepared for labor [42–44].

The findings in this study are useful for policymakers responsible for increasing the number of NHI participants, especially pregnant women. The analysis results provide a precise policy target: pregnant women who are low educated, poor, primiparous, and have inadequate knowledge of pregnancy’s danger signs.

The government needs to accelerate the intensity of awareness campaigns towards increasing the proportion of pregnant women who participate in the NHI program. The government can carry out interventions focusing on efforts to increase the understanding of pregnant women about pregnancy and childbirth risks, thereby encouraging them to protect themselves with health insurance [12, 45].

### Study limitation

This study was conducted by processing data obtained from a previous cross-sectional survey. The authors were unable to confirm the temporal relationship between the exposure variable and the outcome. The study did not investigate several known factors from previous research that investigated determinants of health insurance ownership, including income, smoking behavior, and history of chronic disease [19–21].

## Conclusions

Based on the research results, the study concluded that four variables could explain the determinants of owning health insurance in Indonesia. The four variables were education level, wealth status, parity, and knowledge of the danger signs of pregnancy.

## Data Availability

The author cannot publicly share the data because a third party and the ICF who own the data do not have permission to share it. The 2017 IDHS data set name requested from the ICF (data set of childbearing age women) is available from the ICF contact https://dhsprogram.com for researchers who meet the criteria for access to confidential data.

## Abbreviations

ANC: Antenatal Care
NHI: National Health Insurance
IDHS: Indonesia Demographic and Health Survey
ICF: Inner City Fund
VIF: variance inflation factor

## References

[1] Laksono AD, Rukmini R, Wulandari RD (2020). Regional disparities in antenatal care utilization in Indonesia. PLoS One.

[2] Sanogo NA, Yaya S (2020). Wealth status, health insurance, and maternal health care utilization in Africa: evidence from Gabon. Biomed Res Int.

[3] Anindya K, Lee JT, McPake B, Wilopo SA, Millett C, Carvalho N (2020). Impact of Indonesia’s national health insurance scheme on inequality in access to maternal health services: a propensity score matched analysis. J Glob Health.

[4] Achadi EL. Maternal and Neonatal Death in Indonesia (Kematian Maternal dan Neonatal di Indonesia) [Internet]. Rakerkernas 2019. Jakarta; 2019. Available from: http://www.depkes.go.id/resources/download/info-terkini/rakerkesnas-2019/SESII/Kelompok1/1-Kematian-Maternal-dan-Neonatal-di-Indonesia.pdf.

[5] Masruroh Yusuf A, Rohmah N, Pakki IB, ADP S, Andayani Q, Laksono AD (2021). Neonatal death incidence in healthcare Facility in Indonesia: does antenatal care matter?. Indian J Forensic Med Toxicol.

[6] Utami SM, Handayani F, Hidayah M, Wulandari RD, Laksono AD (2020). Ecological analysis of preeclampsia/eclampsia case in Sidoarjo regency, Indonesia, 2015-2019. Indian J Forensic Med Toxicol.

[7] National Population and Family Planning Board, Statistics Indonesia, Ministry of Health, & The DHS Program. (2018). *The 2017 Indonesia demographic and health survey*. Jakarta. Retrieved from https://www.dhsprogram.com/pubs/pdf/FR342/FR342.pdf

[8] Suparmi, Barida I, Maisya HL (2019). Health insurance as a solution for barriers to maternal healthcare access in Indonesia.

[9] Nasution SK, Mahendradhata Y, Trisnantoro L (2020). Can a National Health Insurance Policy Increase Equity in the utilization of skilled birth attendants in Indonesia? A secondary analysis of the 2012 to 2016 National Socio-Economic Survey of Indonesia. Asia Pac J Public Health.

[10] Wulandari RD, Laksono AD, Matahari R (2020). The effects of health insurance on maternity Care in Health Services in Indonesia. Int J Innov Creativity Change.

[11] Suharmiati Laksono AD, Astuti WD (2013). Policy review on health Services in Primary Health Center in the border and remote area (review Kebijakan tentang Pelayanan Kesehatan Puskesmas di Daerah Terpencil Perbatasan). Bull Health Syst Res.

[12] Sukirman R, Wahyono TYM, Shivalli S (2020). Determinants of healthcare facility utilization for childbirth in Kuantan Singingi regency, Riau province, Indonesia 2017. BMC Public Health.

[13] Yaya S, Da F, Wang R, Tang S, Ghose B (2019). Maternal healthcare insurance ownership and service utilisation in Ghana: analysis of Ghana demographic and health survey. PLoS One.

[14] Aizawa T (2019). The impact of health insurance on out-of-pocket expenditure on delivery in Indonesia. Health Care for Women International.

[15] Abu Bakar A, Samsudin S (2016). Determinants of health care seeking behavior: does insurance ownership matters?. Int J Econ Financ Issues.

[16] Müllerschön J, Koschollek C, Santos-Hövener C, Kuehne A, Müller-Nordhorn J, Bremer V (2019). Impact of health insurance status among migrants from sub-Saharan Africa on access to health care and HIV testing in Germany: a participatory cross-sectional survey 11 medical and health sciences 1117 public health and health services 11 medical and Healt. BMC Int Health Hum Rights.

[17] Wulandari RD, Laksono AD (2020). Are problems during pregnancy a predictor of childbirth in the hospital?: determinants analysis of hospital childbirth in urban poor communities in Indonesia. Indian J Forensic Med Toxicol.

[18] Alo CN, Okedo-Alex IN, Akamike IC (2020). Determinants of willingness to participate in health insurance amongst people living with HIV in a tertiary hospital in south-East Nigeria. Niger Postgrad Med J.

[19] Idris H, Satriawan E, Trisnantoro L (2017). Determinant of health insurance ownership in the informal sector: a panel study from Indonesia family life survey. Adv Sci Lett.

[20] Sari B, Idris H (2019). Determinant of independent national health insurance ownership in Indonesia. Malaysian J Publ Health Med.

[21] Rahmadani S, Marhania Abadi MY, Marzuki DS, Sudirmanb Fajrin MA (2020). Analysis of independent National Health Insurance ownership of informal workers: study of market traders in Gowa District, Indonesia. Enfermeria Clinica.

[22] Mahendra IGAA, Wilopo SA, Sukamdi, Putra IGNE (2019). The role of decision-making pattern on the use of long-acting and permanent contraceptive methods among married women in Indonesia. Eur J Contracept Reprod Health Care.

[23] Miraldo M, Proppera C, Williams RI (2018). The impact of publicly subsidised health insurance on access, behavioural risk factors and disease management. Soc Sci Med.

[24] Nugraheni WP, Mubasyiroh R, Hartono RK (2020). The influence of Jaminan Kesehatan Nasional (JKN) on the cost of delivery services in Indonesia. PLoS One.

[25] Wulandari RD, Laksono AD. Determinants of knowledge of pregnancy danger signs in Indonesia. 2020;15(5):e0232550. 10.1371/journal.pone.0232550. 10.1371/journal.pone.0232550 PMC7239433 32433645

[26] Sisira Kumara A, Samaratunge R (2019). Health insurance ownership and its impact on healthcare utilization: evidence from an emerging market economy with a free healthcare policy. Int J Soc Econ.

[27] Tille F, Röttger J, Gibis B, Busse R, Kuhlmey A, Schnitzer S (2019). Patients’ perceptions of health system responsiveness in ambulatory care in Germany. Patient Educ Couns.

[28] Ipa M, Laksono ADAD, Astuti EPEP, Prasetyowati H, Hakim L (2020). Predictors of malaria incidence in rural eastern Indonesia. Indian J Forensic Med Toxicol.

[29] Laksono AD, Wulandari RD, Efendi F (2020). Determinants of hospital utilisation among urban poor societies in Indonesia. Int J Innov Creativity Change.

[30] Laksono AD, Wulandari RD, Kusrini I, Ibad M (2021). The effects of mother’s education on achieving exclusive breastfeeding in Indonesia. BMC Public Health.

[31] Kusrini I, Fuada N, Supadmi S, Laksono AD (2021). Education as predictor of low birth weight among female worker in Indonesia. Medico-Legal Update.

[32] Megatsari H, Laksono AD, Ibad M, Herwanto YT, Sarweni KP, Geno RAP (2020). The community psychosocial burden during the COVID-19 pandemic in Indonesia. Heliyon.

[33] Laksono AD, Wulandari RD. The barrier to maternity Care in Rural Indonesia. J Public Health (Berl). 2020. 10.1007/s10389-020-01274-3.

[34] Rohmah N, Yusuf A, Hargono R, Laksono AD, Masruroh I, I., & Walid, S. (2020). Determinants of teenage pregnancy in Indonesia. Indian J Forensic Med Toxicol.

[35] Kusi A, Fenny A, Arhinful DK, Asante FA, Parmar D (2018). Determinants of enrolment in the NHIS for women in Ghana – a cross sectional study. Int J Soc Econ.

[36] Williams GA, Parmar D, Dkhimi F, Asante F, Arhinful D, Mladovsky P (2017). Equitable access to health insurance for socially excluded children? The case of the National Health Insurance Scheme (NHIS) in Ghana. Soc Sci Med.

[37] Wen C, Wallace JL (2019). Toward human-centered urbanization? Housing ownership and access to social insurance among migrant households in China. Sustainability (Switzerland).

[38] Kunpeuk W, Teekasap P, Kosiyaporn H, Julchoo S, Phaiyarom M, Sinam P (2020). Understanding the problem of access to public health insurance schemes among cross-border migrants in Thailand through systems thinking. Int J Environ Res Public Health.

[39] Zhang J, Mou Y, Liao J, Xiong H, Duan Z, Huang Y (2019). Uptake of maternal care and childhood immunization among ethnic minority and Han populations in Sichuan province: a study based on the 2003, 2008 and 2013 health service surveys. BMC Pregnancy Childbirth.

[40] Wulandari RD, Laksono AD (2020). Is parity a predictor of neonatal death in Indonesia? Analysis of the 2017 Indonesia demographic and health survey. Indian J Forensic Med Toxicol.

[41] Laksono AD, Wulandari RD (2020). Understanding neonatal death in urban area in Indonesia. Medico-Legal Update.

[42] Vijay NR, Kumare B, Yerlekar DS (2015). Awareness of obstetric danger signs among pregnant women in tertiary care teaching hospital. J SAFOG.

[43] Woldeamanuel GG, Lemma G, Zegeye B (2019). Knowledge of obstetric danger signs and its associated factors among pregnant women in Angolela Tera District, northern Ethiopia. BMC research notes.

[44] Wassihun B, Negese B, Bedada H, Bekele S, Bante A, Yeheyis T (2020). Knowledge of obstetric danger signs and associated factors: a study among mothers in Shashamane town, Oromia region, Ethiopia. Reprod Health.

[45] Belay HG, Limenih MA (2020). Intents of women on obstetric danger signs and its associated factors in Farta Woreda, Ethiopia, 2017. J Health Care Poor Underserved.

